# A Systematic Review on Detraining Effects after Balance and Fall Prevention Interventions

**DOI:** 10.3390/jcm10204656

**Published:** 2021-10-11

**Authors:** Shaghayegh Modaberi, Esmaeel Saemi, Peter A. Federolf, Steven van Andel

**Affiliations:** 1 Department of Sport Sciences, Faculty of Social Sciences, Imam Khomeini International University, Qazvin 34149-16818, Iran; modaberi@soc.ikiu.ac.ir; 2 Department of Motor Behavior and Sport Psychology, Shahid Chamran University of Ahvaz, Ahvaz 83151-61355, Iran; e.saemi@scu.ac.ir; 3 Department of Sport Science, University of Innsbruck, 6020 Innsbruck, Austria; Peter.Federolf@uibk.ac.at

**Keywords:** postural control, balance, detraining, falls, inactivity, lockdown, COVID-19, ageing

## Abstract

Since the COVID-19 pandemic hit, lockdowns have been implemented to fight off infections in countries around the world. Whilst this measure is without a doubt effective against spreading infection, it might also decrease participation in exercise. For older adults, exercise is particularly important in the prevention of falls, and sudden detraining because of a lockdown or due to other causes might have detrimental consequences. This systematic review study aims to assess what is currently known on detraining effects for balance outcomes. Nine studies were included within this review. Results suggest that detraining effects could already be significant as early as 4 weeks after stopping the intervention. Programs that specifically focus on improving balance were more robust against detraining, with most positive effects still being present after 8 weeks. However, even with a specific focus on balance, studies started to show some signs of detraining. The current study is limited by the low number of included studies in the review, indicating a need to further confirm these results.

## 1. Introduction

Around the world, populations are ageing [1], leading to an increased incidence of age-related accidental injuries, often caused by falls [2,3]. In recent years, evidence has been building that falls, particularly in older individuals, are to some degree preventable. Specifically, several studies confirmed that exercise programs can be effective in improving balance and in lowering fall rates in older adults, as summarized in recent review papers [4,5]. However, recently, access to exercise programs has been severely limited by the global response to the COVID-19 pandemic: sports facilities were closed, and in some cases, citizens were asked to stay at home, sometimes for periods of several weeks. Since older adults have a higher risk for a difficult progression in case of a COVID-19 infection, this age group is particularly subjected to restrictions. This is unfortunate, since particularly in this age group, exercises have a number of tangible health benefits, including the maintenance of the balance and postural control skills that are needed to prevent falls and accidental injuries. 

The concept of detraining has been described in previous literature as “the partial or complete loss of training-induced anatomical, physiological, and performance adaptations, as a consequence of training reduction or cessation” [6], p. 80. Historically, research has mainly focused on the physiological effects of detraining after strength or resistance training, e.g., [7,8], but very little is known about detraining in balance skill [9]. The current study assumes that a sudden stop in balance training might induce detraining effects that could increase the risk of falls in older adults. While the recently experienced lockdowns due to COVID-19 motivated the current study, our research question is of general interest, since a sudden stop of participation in a regular exercise program can occur for various reasons. As such, the current study will focus on the “performance adaptation” component of detraining to assess the effects of a cessation of balance training for indicators of fall risk. 

The aim of the current study was to review the available scientific literature on detraining effects after a sudden stop of an exercise program on outcomes related to fall risk. To operationalize this aim, we shall consider fall risk in two ways. Firstly, as a direct measure of fall risk, studies reporting on fall rates would be accepted into the review. Second, as an indirect measure, studies reporting on fall risk assessments will be accepted. This second definition might pose some methodological challenge, since indirect measures of fall risk are numerous [10] and might not always be easily compared. For these studies, the study characteristics shall be synchronized in one table in an effort to distill common principles from the literature.

## 2. Materials and Methods

This systematic review was carried out in accordance with the Preferred Reporting Items for Systematic Reviews guideline [11] and under PROSPERO registration number CRD42020199932.

### 2.1. Information Sources and Search Strategy

The initial search was performed on 20 August 2020, by using the following databases: Web of Science (all databases), Scopus, PubMed, PEDro, and Cochrane Library. Searches were conducted in English. The option “advanced searches” was chosen for searches in Web of Science, PubMed, and Scopus with regard to the articles’ title, abstract, and keywords. A simple search was done in Cochrane library and PEDro. The Boolean operators used were “OR” within the construct, and “AND” between constructs. The following search terms were used:

(detraining OR inactivity)

AND

(balance OR **“**postural control**”** OR **“**postural stability**”**)

AND

(**“**fall risk**”** OR **“**falls risk**”** OR falling OR **“**fall rate**”**)

### 2.2. Eligibility Criteria and Study Selection

Articles were eligible for inclusion in the current study according to the following criteria. Any type of intervention study was accepted in the review (e.g., randomized controlled trials, non-randomized controlled trials, pre-post studies with no control group). Only studies written in English were considered. In terms of outcomes, only studies that measured balance control in humans after a period of detraining were included. Here, we consider any study that reports on the effects of a sudden stop to exercise participation to fit this description. No limitations were set to the characteristics of the participants (e.g., young or old, athletes or sedentary), with the exception that clinical groups in which balance might be affected (e.g., amputations, neurological pathology, recent surgery) were excluded from the review. No limitations were set for the year of publishing. 

In the first screening step, duplicates were removed using EndNote software. Further screening steps were performed after exporting to Microsoft Excel. Two authors were responsible for the screening process (S.M. and E.S.) and screened first the titles and later the abstracts and, finally, on the full text to sort out papers based on the inclusion criteria. 

### 2.3. Data Extraction

Two authors were responsible for data extraction (S.M. and S.A.). The following factors were extracted for any study that was included in the present review: study design, training duration, detraining duration, measurement tools, groups, age of participants, study results with respect to effect of training, effect of detraining, conclusion, and future recommendations.

### 2.4. Quality Assessment

Methodological quality in all studies was assessed using the Crowe Critical Appraisal Tool (or CCAT; [12,13]). Since several study designs were eligible, it was important to use a quality assessment tool designed for a broad range of study designs. The CCAT was developed to assess methodological quality of randomized controlled trials as well as other study designs and was, therefore, suitable for the current study. We shall interpret CCAT scores in quintiles to categorize studies as very low (0–8 points), low (9–16 points), moderate (17–24 points), high (25–32 points) or very high (33–40 points) methodological quality similar to [14]. The current review reports both the section scores per paper as well as the total scores overall, which are needed to draw conclusions both on an individual paper level, as well as for the entire field. 

## 3. Results

**Figure 1:**
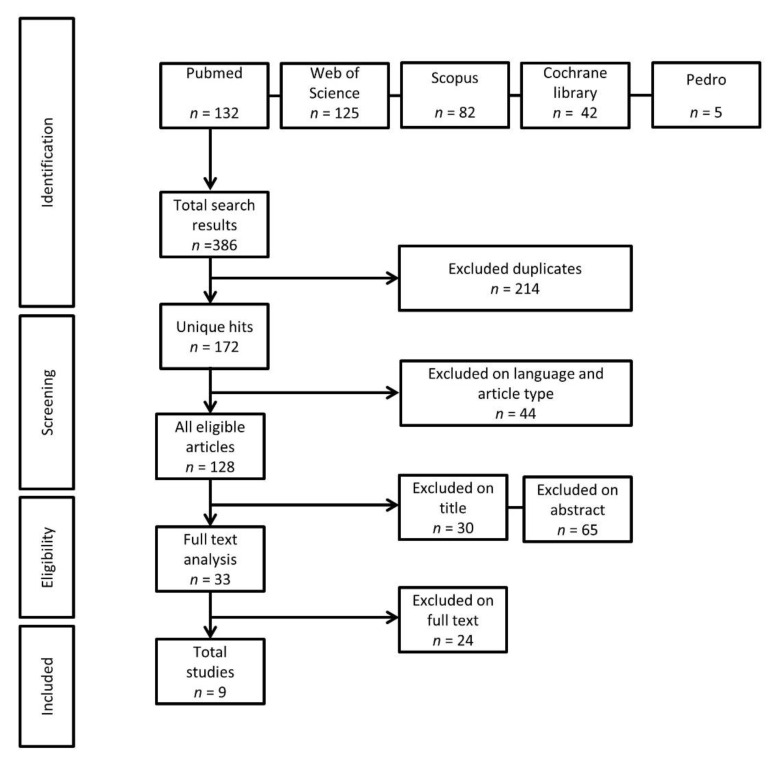
Flow diagram outlining the article selection steps.

From the systematic search, 386 papers were identified as potentially eligible for inclusion. Following the article selection steps (Figure 1), nine papers met the criteria for inclusion in the current study. No studies were identified that reported on fall rates after a detraining period, and as such, only studies that reported on indirect measures of fall risk were included. Characteristics of the included papers are displayed in Table 1.

### 3.1. Effects of Detraining

From the originally identified papers, only nine clearly described the effects of detraining on the postural control system. The duration of the detraining or retention period ranged from 4 weeks (*n* = 1 paper) to 6 weeks (*n* = 1), 8 weeks (*n* = 3), 12 weeks (*n* = 2), 60 days (*n* = 1), and the highest reported duration of detraining was half a year (*n* = 1). One included study did not report on detraining from an intervention, but rather “detraining from everyday life” by administering 60 days of bed rest. All of the other papers provided details of an intervention with a length of 8 weeks (*n* = 1), 10 weeks (*n* = 1), 12 weeks (*n* = 2), 16 weeks (*n* = 3) or 40/80 weeks (*n* = 1, in this particular study, participants could choose their own intervention length to increase adherence; [19]). Details of the interventions’ main focus are displayed in Table 1. 

Due to the differences in outcome measures and lengths of detraining, it is futile to quantify exact effects of detraining on a week-by-week basis. This is further complicated by studies that did not report on their effects directly post intervention, so it is unclear whether their detraining values later on indicate significant declines after the exercises were ceased (i.e., [17]). The first clear sign of detraining in all studies was after 4 weeks, at which point the improved plantar flexion proprioception after an 8-week brisk walking program had disappeared [23]. Further, at 6 weeks, the effects of an 8-week whole body vibration training program seemed to have returned to baseline [15]. More exercise effects seemed to disappear between 8 and 12 weeks, after which most benefits from a low-intensity balance program [21] and from seated or weight-bearing resistance training [22] had significantly digressed. 

Exercises for which lasting benefits were reported after specified detraining periods were, firstly, an 8-week aquatic balance training (both with and without combining it with vibration training; outcome measures: 5 times sit-to-stand (STS) and timed up and go (TUG) performance; after 8 weeks of detraining [15]). Second, 16 weeks of Tai Chi still had positive effects on one-leg stance performance (strongest effects on eyes open condition [20] and on ankle plantar and dorsal flexion proprioception [23]) after 8 weeks of detraining. Additionally, third, 12 weeks of functional weightbearing exercise still had a positive effect on “coordinated stability” (the ability to draw a line within the lines of a track, using a pen fixed by a rod to the person at waist level) after 12 weeks of detraining [22]. However, in terms of this last result, it should be noted that outcomes for a more common indicator of fall risk, the “Physiological Profile Assessment”, no longer showed differences compared to controls after 12 weeks of detraining [22]. 

In terms of long-term effects, it is interesting to note the benefits of a strength training protocol reported by Sherk et al. [19] on maximum strength outputs. They showed that, after 6 months of detraining, improvements were better retained if participants had been engaged with the program for a longer period of time (80 vs. 40 weeks of training). Finally, the results of Ritzmann et al. [18] are of relevance. They showed that the effects of detraining due to bed rest can be offset by the introduction of 3-min high-intensity jumping workouts. 

### 3.2. Quality Assessment

Results from the quality assessment are depicted in Table 2. With an average score of 30.1/40, the studies were generally of high quality (one study was categorized as having moderate quality, six studies were categorized as having high quality, and two studies were categorized as having very high methodological quality). On average, the lowest scores (with also the biggest spread) were recorded in the sampling category, which relates to reporting of the sampling method, sample size, and recruitment protocols. 

## 4. Discussion

The current study aimed to assess current literature on detraining effects after discontinuing an exercise program on indicators of fall risk. Through our systematic review protocol, a total of nine studies were considered eligible for inclusion in this study. Four studies showed effects that seemed relatively robust against the effects of detraining [15,20,22,23]. Common among these four seems to be a specific focus on balance training, in the form of aquatic balance training [15], weight-bearing (as opposed to seated) exercise [22], and Tai Chi [20,23]. However, programs that did not specifically target balance, or did so on low intensity, seem less effective after detraining (i.e., brisk walking, whole body vibration training, low-intensity balance training, seated or weight-bearing resistance training). No studies were found that assessed detraining effects directly on fall prevalence. Indirect effects on fall risk can be inferred from the included studies, considering that several of the analyzed outcome variables, e.g., one leg standing time [24] or the timed up and go (TUG) test [25] show good association to the actual fall risk. However, in this context, it should also be noted that not all recent studies confirm a strong association between these performance variables and predictive power as fall risk indicators [26].

It might be argued that bedrest is conceptually different than detraining and, therefore, should not be included in this review. We do not seek to discuss the definition of the concept of detraining in this study and emphasize that out operationalization of this concept (“any study that reports on the effects of a sudden stop to exercise participation”) is just one way to work with this concept. This definition was adhered to because even studies that might not fit the strictest definition of detraining might illustrate important mechanisms for fall prevention in a practical sense. Even though a lock down is not as severe as a bedrest prescription, for older adults who are in risk groups (and may not dare to go outside), this measure will still result in severe increases in sedentary behavior. It is, therefore, promising to see that the effects of bedrest can be offset by even very short bouts of intense exercise [18]. However, the nature of the introduced program with its focus on unstable jumping exercises might not be the best fit for the older adult community where, due to age-related loss of bone mass [27], any fall might lead to the breaking of bones. As such, future studies should investigate how this training program could be adapted to keep older adults fit and steady at home. 

The results from the quality assessment showed that the methodological quality of the articles overall was high, with a high variability of scores in the “sampling category”. Future studies in this field would do well to provide a detailed description of their sampling strategy, for instance, by including details on a recruitment strategy, sample size calculation, and target population. 

It is interesting that the current study set no restrictions to the age of included participants, and yet, all but one study showed a sample aged 62 years or older. This was most likely caused by the inclusion of search terms related to fall risk, as this is commonly assessed using an older cohort. This does limit the generalizability of our results to say most about this older cohort. The generalizability of the results is further limited by the fact that detraining might occur for older adults who regularly participate in physical activity, but it should be noted that, in general, this cohort is known to show high levels of sedentary behavior [28]. 

One inherent limitation to the study’s design is that our search might not have identified all studies done on detraining, as some authors might not have specifically used this term in their study’s protocol. That is, if a study describes an intervention with pre-test, post-test, and a relatively late retention test, then this could potentially be a useful addition to the current study. However, this is difficult to capture in a systematic search, as terms related to the word “retention” are not specific to this experimental design and significantly increase the number of hits towards the unmanageable. As such, it was decided to specifically focus the search on studies that mention the terms “detraining” or “inactivity” in order to achieve more focused set of search results. This has led to the inclusion of only nine studies, which might limit the applicability of the current results. These results should be further confirmed in future studies before drawing strong conclusions in regards to detraining and fall prevention. The current study can be used to guide future studies in this field. Specifically, future studies could hypothesize that training with a challenge to balance might be less susceptible to detraining effects compared to general training and that training effects would be sustained for about 8 weeks. 

The current study provides preliminary evidence for the sustained effects of balance training 8 weeks after cessation of specific balance training. However, at this 8-week timepoint, even in the more successful programs, there were outcome variables that started to show significant reductions in performance. It could, thus, be expected that detraining would also start to affect participants in the more successful programs after 8 weeks. Should these results be confirmed in future studies, then it would imply that restrictions that limit the availability of balance training programs might have a negative effect on fall rates for older adults that regularly participate in fall prevention exercise, if they are longer than 8 weeks. 

## Figures and Tables

**Table 1:** Data extraction table collating the characteristics from the (*n* = 9) included studies.

	Participants (N at Study Onset)	Training Duration	Training Intensity	Detraining Duration	Type of Intervention	Outcome Measures	Effect of Training	Effect of Detraining	Notes
Abbasi et al. [15]	Age: 70 ± 9.6 years (*n* = 60)	8 weeks	3 × 60 min per week	8 weeks, measured at 4, 6, and 8 weeks	Aquatic balance (AB) training	5 × STS	Significantly improved	Improvements retained at 8 w	Attrition information: No mention of dropout rate
						TUG	Significantly improved	Improvements retained at 8 w	
					Whole-body vibration (WBV) training	5 × STS	Significantly improved	*Returned to baseline at 6 w detraining*	
						TUG	Significantly improved	*Returned to baseline at 6 w detraining*	
					Combined AB and WBV	5 × STS	Significantly improved	Improvements retained at 8 w	
						TUG	Significantly improved	Improvements retained at 8 w	
Ansai et al. [16]	Age: 82.4 ± 2.4 years (*n* = 69)	16 weeks	3 × 60 min per week	6 weeks	Multicomponent training, containing aerobic, strength, and balance component	5 × STS	No significant improvement		Effects following “intention to treat” principle. Some effects were found in “high adherence” group. Attrition information: *n* = 1 participant dropped out before the post-test and 3 were lost to follow-up.
						Balance tests	No significant improvement		
						TUG	No significant improvement		
					Resistance training	5 × STS	No significant improvement		
						Balance tests	No significant improvement		
						TUG	No significant improvement		
Harvey et al. [17]	Age: 78.4 ± 6.9 years (*n* = 23)	10 weeks	3 × 40 min total	4 weeks	Motivational interview and retrospective feedback, one group with and one group without real time feedback	Chair sit and reach		*No significant improvement*	Only main effects between baseline and retention test reported. No effects of intervention itself. Attrition information: *n* = 13 participants completed the program.
						30 s STS		Significantly improved	
						BST		*No significant improvement*	
						TUG		Significantly improved	
						Sedentary behavior		*No significant improvement*	
Ritzmann et al. [18]	Age: 30 ± 7 years (*n* = 23)			60 days head tilted down bedrest	Full bedrest	One-leg stance sway		*30–105% decrease*	No training intervention/detraining study, but study showing effects of bedrest on healthy subjects. Attrition information: one participant reallocated to full bedrest for medical reasons. *n* = 0 participants dropped out.
						One-leg stance co-contraction		*Increased co-contraction*	
						10 m walk test		*Significant decreases*	
						TUG		*20–40% decrease*	
						10 × STS		*20–80% decrease*	
					Bedrest with in total 48–3 min jump sessions	One-leg stance sway		No significant decrease	
						One-leg stance co-contraction		No significant decrease	
						10 m walk test		Most variables unchanged	
						TUG		No significant decrease	
						10 × STS		No significant decrease	
Sherk et al. [19]	Age: 64.5 ± 0.5 years (*n* = 69)	40 or 80 weeks	2 or 3 times per week	6 months	Resistance training 40 weeks	Upper body 1RM strength	52% increase	*About 15% decrease*	Detraining values visually assessed from Figure 3 in [19]. Attrition information: *n* = 69 participants returned for 6 month follow-up.
						Lower body 1RM strength	71% increase	*About 9% decrease*	
					Resistance training 80 weeks	Upper body 1RM strength	22% increase	*About 4% decrease*	
						Lower body 1RM strength	33% increase	No visible change	
Sun et al. [20]	Age: 64.2 ± 3.18 years (*n* = 48)	16 weeks	5 × 60 min times per week	8 weeks	Tai Chi	Eyes open single leg stance variables	7/7 variables significantly improved	7/7 variables remain significantly different from baseline after 8 w detraining	No intention to treat data. Only participants who completed the program are reported 25% drop outs not. Reporte dattrition information: *n* = 36 participants completed the study.
						Eyes closed single leg stance variables	7/7 variables significantly improved	5/7 variables remain significantly different from baseline after 8 w detraining	
					Brisk Walking	Eyes open single leg stance variables	7/7 variables significantly improved	4/7 variables remain significantly different from baseline after 8 w detraining	
						Eyes closed single leg stance variables	7/7 variables significantly improved	5/7 variables remain significantly different from baseline after 8 w detraining	
Toulotte et al. [21]	Faller-group age: 71.1 ± 5.0 years (*n* = 16)	12 weeks	2 × 60 min per week	12 weeks	Low-intensity balance program	30 s one-leg stance test eyes open	Significantly improved	*Significant decrease*	Attrition information: No mention of dropout rate.
						30 s one-leg stance test eye closed	Significantly improved	*Significant decrease*	
						Gait variables single task	5/5 variables significantly improved	*0/5 variables stay on post-intervention value after 12 w detraining*	
						Gait variables dual task	5/5 variables significantly improved	*1/5 variables stay on post-intervention value after 12 w detraining*	
	Non-faller-group age: 68.4 ± 4.5 years (*n* = 8)	12 weeks	2 × 60 min per week	12 weeks	Low-intensity balance program	30 s one-leg stance test eyes open	Significantly improved	*Significant decrease*	
						30 s one-leg stance test eyes closed	No significant improvement	*Significant decrease*	
						Gait variables single task	5/5 variables significantly improved	*1/5 variables stay on post-intervention value after 12 w detraining*	
						Gait variables dual task	5/5 variables significantly improved	*0/5 variables stay on post-intervention value after 12 w detraining*	
Vogler et al. [22]	Age: 80 ± 7 years (*n* = 180 in 3 groups)	12 weeks	3 times per week	12 weeks	Weight-bearing functional exercise	Physiological Profile Assessment	Visibly improved compared to controls	*Not different from controls at 12 w detraining*	Control condition: social visits. Direct post-intervention data not reported, differences reported here are visually assessed from Figure 2 in [22]. Attrition information: no difference in adherence between groups, *n* = 9 participants dropped out before the end of the 12 w training phase and *n* = 6 participants were lost to the 24 w follow-up.
						Maximal balance range	Visibly improved compared to controls	*Not different from controls at 12 w detraining*	
						Coordinated stability	Visibly improved compared to controls	Significantly improved compared to controls	
					Seated resistance exercise	Physiological Profile Assessment	Visibly improved compared to controls	*Not different from controls at 12 w detraining*	
						Maximal balance range	Visually not different from controls		
						Coordinated stability	Visually not different from controls		
Zhang et al. [23]	Age: 62.01 ± 4.40 years (*n* =60)	16 weeks	5 × 60 min times per week	8 weeks	Tai Chi	Plantar flexion proprioception	Significantly improved	Improvements retained at 8 w	Attrition information: *n* = 52 participants completed the study.
						Dorsal flexion proprioception	Significantly improved	Improvements retained at 8 w	
						Inversion proprioception	No significant improvement compared to baseline		
						Eversion proprioception	No significant improvement compared to baseline		
					Brisk walking	Plantar flexion proprioception	Significantly improved	*Returned to baseline at 4 w detraining*	
						Dorsal flexion proprioception	No significant improvement compared to baseline		
						Inversion proprioception	No significant improvement compared to baseline		
						Eversion proprioception	No significant improvement compared to baseline		

Notes: STS = “sit to stand”, TUG = “timed up and go”, BST = “balance screening tool”, 1RM = “one repetition maximum”, w = weeks. Results that show significant detraining are displayed in italics.

**Table 2:** Results of the CCAT quality assessment.

	Preliminaries	Introduction	Design	Sampling	Data Collection	Ethical Matters	Results	Discussion	Total
Abbasi et al. [15]	3	4	4	0	2	3	3	3	22 ^M^
Ansai et al. [16]	3	4	5	4	4	4	4	3	31 ^H^
Harvey et al. [17]	3	3	5	2	5	5	3	4	30 ^H^
Ritzmann et al. [18]	4	5	5	4	5	5	5	4	37 ^VH^
Sherk et al. [19]	4	4	2	3	5	5	3	3	29 ^H^
Sun et al. [20]	2	2	4	3	5	5	5	4	30 ^H^
Toulotte et al. [21]	4	3	5	3	3	4	3	5	30 ^H^
Vogler et al. [22]	3	5	3	5	5	5	3	4	33^V H^
Zhang et al. [23]	4	4	4	3	4	4	4	2	29 ^H^
Mean values	3.3	3.8	4.1	3.0	4.2	4.4	3.7	3.6	30.1
Standard deviation	0.71	0.97	1.05	1.41	1.09	0.73	0.87	0.88	3.95

Note. Categorization of methodological quality of papers is noted in superscript ranging from moderate (^M^) to high (^H^) and very high (^VH^).

## Data Availability

All data used in the current study re available in the public domain.
